# Increased Autophagy in Placentas of Intrauterine Growth-Restricted Pregnancies

**DOI:** 10.1371/journal.pone.0040957

**Published:** 2012-07-16

**Authors:** Tai-Ho Hung, Szu-Fu Chen, Liang-Ming Lo, Meng-Jen Li, Yi-Lin Yeh, T’sang-T’ang Hsieh

**Affiliations:** 1 Department of Obstetrics and Gynecology, Chang Gung Memorial Hospital at Taipei, Taipei, Taiwan; 2 Department of Chinese Medicine, College of Medicine, Chang Gung University, Taoyuan, Taiwan; 3 Department of Physical Medicine and Rehabilitation, Cheng Hsin Rehabilitation Medical Center, Taipei, Taiwan; Virgen Macarena University Hospital, School of Medicine, Spain

## Abstract

**Background:**

Unexplained intrauterine growth restriction (IUGR) may be a consequence of placental insufficiency; however, its etiology is not fully understood. We surmised that defective placentation in IUGR dysregulates cellular bioenergic homeostasis, leading to increased autophagy in the villous trophoblast. The aims of this work were (1) to compare the differences in autophagy, p53 expression, and apoptosis between placentas of women with normal or IUGR pregnancies; (2) to study the effects of hypoxia and the role of p53 in regulating trophoblast autophagy; and (3) to investigate the relationship between autophagy and apoptosis in hypoxic trophoblasts.

**Methodology/Principal Findings:**

Compared with normal pregnant women, women with IUGR had higher placental levels of autophagy-related proteins LC3B-II, beclin-1, and damage-regulated autophagy modulator (DRAM), with increased p53 and caspase-cleaved cytokeratin 18 (M30). Furthermore, cytotrophoblasts cultured under hypoxia (2% oxygen) in the presence or absence of nutlin-3 (a p53 activity stimulator) had higher levels of LC3B-II, DRAM, and M30 proteins and increased Bax mRNA expression compared with controls cultured under standard conditions. In contrast, administration of pifithrin-α (a p53 activity inhibitor) during hypoxia resulted in protein levels that were similar to those of the control groups. Moreover, cytotrophoblasts transfected with LC3B, beclin-1, or DRAM siRNA had higher levels of M30 compared with the controls under hypoxia. However, transfection with Bcl-2 or Bax siRNA did not cause any significant change in the levels of LC3B-II in hypoxic cytotrophoblasts.

**Conclusions/Significance:**

Together, these results suggest that there is a crosstalk between autophagy and apoptosis in IUGR and that p53 plays a pivotal and complex role in regulating trophoblast cell turnover in response to hypoxic stress.

## Introduction

Autophagy is a catabolic process that involves the invagination and degradation of cytoplasmic components through a lysosomal pathway [1]. During autophagy, proteins and organelles are sequestered into double-membrane vesicles called autophagosomes. Autophagosomes ultimately fuse with lysosomes to generate single-membrane autophagolysosomes that mediate the degradation of their contents. Degradation of the sequestered material generates nucleotides, amino acids, and free fatty acids that are recycled for macromolecular synthesis and ATP generation. Low basal levels of autophagy contribute to organelle turnover and bioenergic management. Autophagy is rapidly upregulated under conditions, such as starvation, growth factor deprivation, and hypoxia, when cells need to generate intracellular nutrients and energy [2]. Autophagy is also involved in removing damaged mitochondria or other organelles and degrading intracellular pathogens and protein aggregates that are too large to be removed by the ubiquitin-proteasomal system [3]. These functions of autophagy favor the adaptation of cells and could promote cellular survival during aging, infectious diseases, and neurodegenerative processes.

Unexplained intrauterine growth restriction (IUGR), defined as a failure of the fetus to reach its genetic growth potential, may be a consequence of placental insufficiency. Although its etiology is not fully understood, the most widely recognized predisposing factor for IUGR is deficient invasion of the endometrium by extravillous cytotrophoblasts during the first trimester of pregnancy [4], which leads to incomplete conversion of the spiral arteries such that the myometrial segments do not dilate and remain contractile. Furthermore, the spiral arteries in women with IUGR often display thrombosis and acute atherosis. These secondary changes lead to a significant reduction in the caliber of the vessels. As a result, perfusion of the intervillous space is impaired, leading to the assumption that placental changes associated with IUGR, such as decreased villous branching, a reduction in the volume and surface area of terminal villi, and aberrant cell turnover in villous trophoblasts [5], arise from a state of chronic hypoxia. In fact, hypoxia has been shown to cause trophoblast dysregulation and loss of functional mass of the villous trophoblast via several mechanisms, including apoptosis [6], mitochondrial oxidants [7], and the stress response of the endoplasmic reticulum [8]. Evidence of these insults has been characterized in the placentas from women with IUGR [9].

Increased p53 activity is one of several cell death pathways that regulate apoptosis in villous trophoblasts [10]. Compared with women with normal pregnancies, women with IUGR have higher levels of placental p53, and the association between altered trophoblast cell turnover in IUGR and increased p53 expression is reminiscent of that following exposure to hypoxia [11], [12]. Recent studies from other organ systems also suggest that p53 plays an important role in the regulation of autophagy [13]. However, the interactions between apoptosis and autophagy mediated through the p53 pathway in human trophoblasts remain unclear [14].

**Table 1:** Characteristics of the study population.

	Normal pregnancy(n = 14)	IUGR (n = 14)	Preeclampsia (n = 18)	Preeclampsia with IUGR (n = 15)
Age (y)	33.4±1.4	33.1±2.9	32.7±6.0	33.4±3.5
Primiparity	11 (79%)	12 (86%)	15 (83%)	11 (73%)
Pre-pregnancy BMI (kg/m^2^)	22.3±3.8	21.1±4.6	25.4±4.4^a^	24.1±4.7
Blood pressure before delivery				
Systolic (mm Hg)	123±14	122±12	158±16^c^	163±25^c^
Diastolic (mm Hg)	77±10	74±10	97±15^a^	102±20^b^
Urine protein (mg/dL)	0	0 (0–500)	100 (30–1000)^b^	500 (30–1000)^c^
Hemoglobin (g/dL)	11.9±1.5	12.1±1.6	12.5±2.4	12.4±1.5
Platelet count (10^3^/ µL)	231 (42–292)	218 (147–362)	210 (92–410)	210 (126–369)
Gestational age (wk)	38.3±1.0	38.1±1.2	37.4±2.8	35.2±3.4^a^
Birth weight (g)	3176±567	2284±378^a^	2862±658	1855±525^c^
Placental weight (g)	715±188	457±97^b^	572±136	439±123^c^
1-minute Apgar score	9 (7–9)	9 (7–9)	9 (7–9)	9 (6–9)
5-minute Apgar score	10 (9–10)	10 (8–10)	10 (9–10)	10 (7–10)

BMI  =  body mass index; IUGR  =  intrauterine growth restriction.

Data presented as the means (± SD), medians (range) or n (%).

a *P*<0.05;

b *P*<0.01;

c *P*<0.001, compared to normal pregnant women based on one-way analysis of variance followed by Bonferroni’s test or the Kruskal-Wallis test followed by Dunn’s multiple comparison test.

We surmised that defective placentation in IUGR causes derangement of cellular bioenergic homeostasis, thus leading to increased autophagy in the villous trophoblast. We hypothesized that women with IUGR have more extensive autophagy in the placenta compared with normal pregnant women. The aims of this work were (1) to compare autophagy in the placentas from women with normal pregnancies to those with pregnancies complicated by IUGR, preeclampsia (PE), or both (PE+IUGR); (2) to study the levels of p53 and trophoblast apoptosis in the placentas between women with normal and IUGR pregnancies; (3) to examine the effects of hypoxia and reagents that regulate the activity of p53 on trophoblast autophagy; and (4) to investigate the relationship between autophagy and apoptosis in trophoblasts exposed to hypoxia.

## Materials and Methods

This study was approved by the Institutional Review Board of Chang Gung Memorial Hospital, Taiwan (No. 98-3987B). All placental samples were collected after the subjects provided written informed consent. Reagents were purchased from Sigma-Aldrich (St. Louis, MO) unless otherwise indicated.

### Collection of Placental Samples

Recent reports show that delivery mode has an impact on oxidative stress and autophagy in the placenta [15], [16]. Therefore, we collected placentas from 61 women who had elective cesarean deliveries before the onset of labor to compare the levels of autophagic changes between women with normal pregnancies and those with pregnancies complicated by PE, IUGR, or PE+IUGR. The subjects included 14 women with normal pregnancies, 14 patients with IUGR, 18 patients with PE, and 15 patients with PE+IUGR. Table 1 summarizes the characteristics of these 61 women.

PE was defined as blood pressure ≥140/90 mmHg, with proteinuria ≥300 mg in 24 hours or ≥1+ protein on a urine dipstick after 20 weeks of gestation in a previously normotensive woman [17]. IUGR was defined as having a birth weight below the 5th percentile when corrected for gestational age and fetal gender. The percentiles for growth parameters were derived from a reference Taiwanese population [18]. A normal pregnancy was defined as a pregnancy in which the mother had normal blood pressure, an absence of proteinuria, and no medical or pregnancy complications.

**Figure 1:**
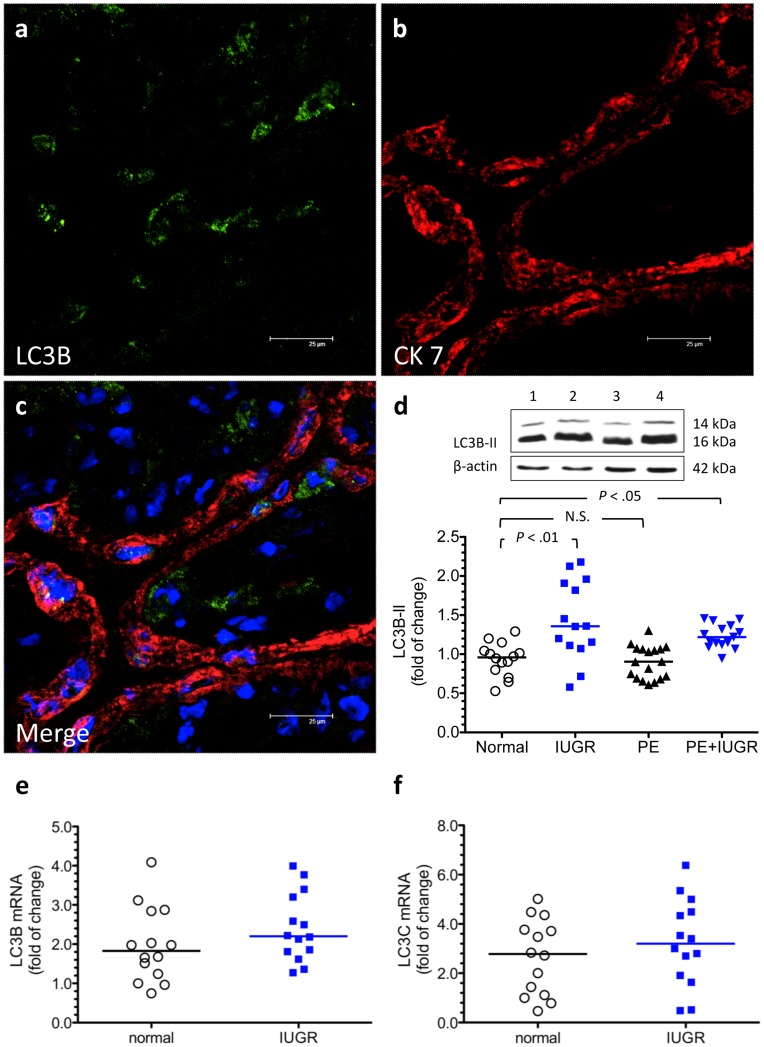
Increased LC3B-II levels in the placentas from pregnancies complicated by IUGR and PE+IUGR. (a–c) Formation of LC3B-punctae was noted in the trophoblasts and stromal cells of IUGR placentas. Scale bar  = 25 µm. (d) An antibody with a stronger reactivity to the type II form of LC3B showed significantly higher placental levels of LC3B-II in women with pregnancies complicated by IUGR (n = 14) and PE+IUGR (n = 15) compared with women with normal pregnancies (n = 14). There was no difference in the levels of placental LC3B-II between women with normal or PE pregnancies (n = 15). Lane 1, normal pregnancy; lane 2, IUGR pregnancy; lane 3, PE pregnancy; and lane 4, PE+IUGR pregnancy. (e, f) There was no difference in the expression of placental LC3B and LC3C mRNA between women with pregnancies complicated by IUGR (n = 14) and those with normal pregnancies (n = 14). Horizontal bars represent the median values. *P* values were based on the Kruskal-Wallis test followed by Dunn’s multiple comparison test (d) or the Mann-Whitney *U*-test (e, f) to compare differences between the groups. PE, preeclampsia; IUGR, idiopathic intrauterine growth restriction; N.S, non-significant.

After the placenta was delivered, five distinct sites were randomly sampled from the maternal side using a transparent sheet bearing a systematic array of sampling windows. Each site was midway between the cord insertion and the periphery of the placenta and midway between the chorionic and basal plates. The placental samples were quickly washed in ice-cold phosphate-buffered saline (PBS) to clear maternal blood, frozen in liquid nitrogen, and stored at −70°C for further processing. All placental samples were collected and processed within 10 minutes after delivery.

### Isolation and Culture of Cytotrophoblasts from Term Placentas

Cytotrophoblasts were isolated from normal term placentas as previously detailed [19]. Briefly, approximately 50 g of villous tissue was collected, finely minced, and dissociated in three 15-minute stages in Hank’s balanced salt solution, 0.25% trypsin (Invitrogen, Carlsbad, CA), and 300 U/mL DNase I. The resulting cell suspension was layered over a 5–70% discontinuous Percoll gradient (Roche Diagnostics GmbH, Mannheim, Germany) and centrifuged at 1200× g for 20 minutes. The cells migrating between the densities of 35 and 50% Percoll were collected and subjected to immunopurification by negative selection over columns consisting of magnetic microbeads coupled to the mouse anti-human HLA-class I antibody. The purified cells were then plated at a minimum of 2×10^5^ cells/cm^2^ in 6-well plates and cultured in a humidified atmosphere with 5% CO_2_ with balanced air (standard conditions). After an overnight rest, the cells were rinsed twice with pre-warmed culture medium to remove the non-attached cells, and the medium was changed every 24 to 48 hours.

**Figure 2:**
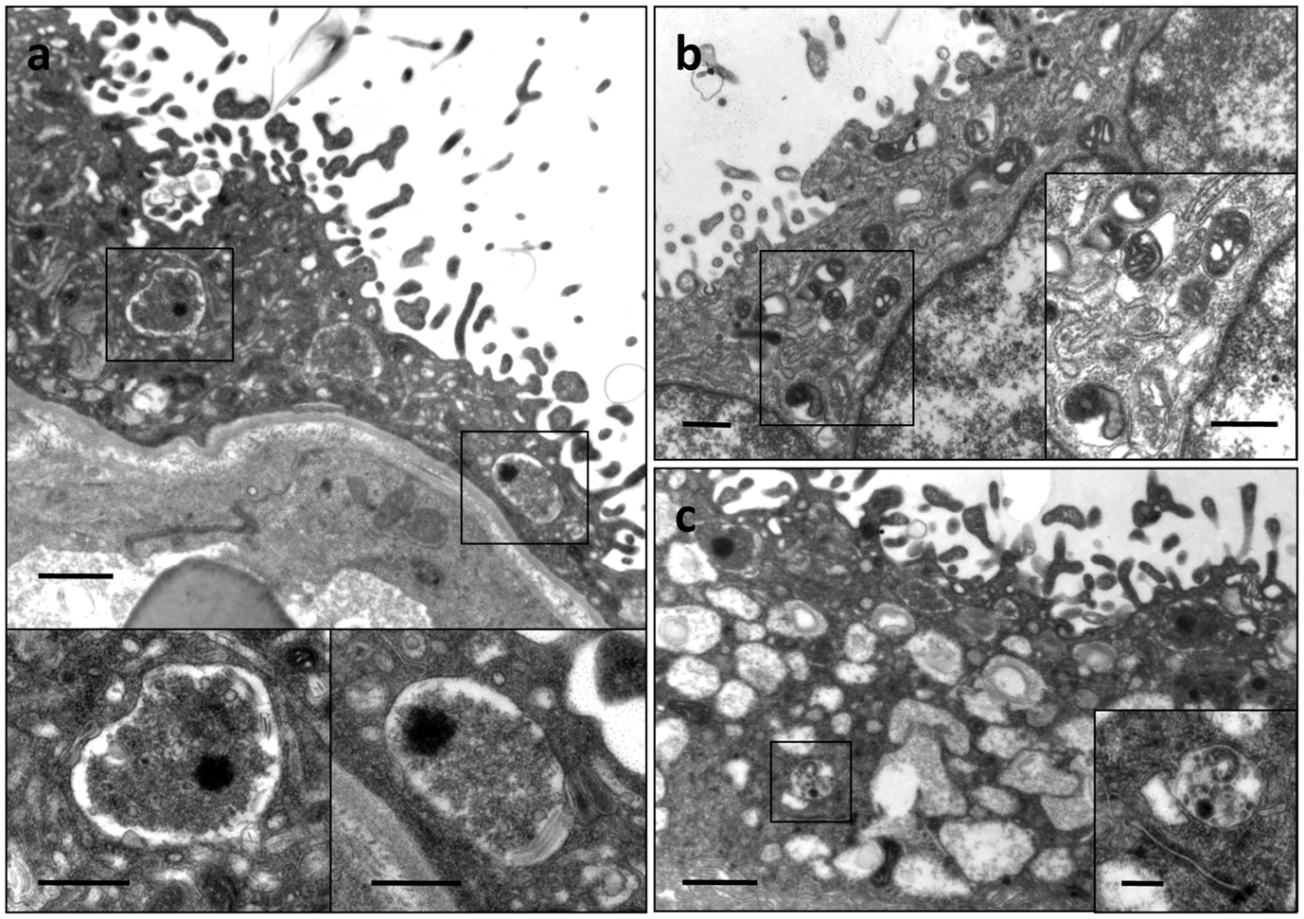
Ultrastructural assessment of autophagic changes in the trophoblast layer of placentas from pregnancies complicated by IUGR and PE+IUGR. Electron micrographs illustrating autophagic vacuoles in the trophoblast layer of the placenta from women with IUGR (a) and PE+IUGR (b, c). These autophagic vacuoles contain intracytoplasmic organelles, such as mitochondria (insets). In addition, loss of mitochondrial integrity (b) and dilatation of cis-ternae of rough endoplasmic reticulum (c) were noted in PE+IUGR placentas. Scale bar  = 1 µm and 500 nm in insets (a, c) and 250 nm and 500 nm in inset (b).

To study the effect of hypoxia and the role of p53 in the regulation of trophoblastic autophagy, cytotrophoblasts were cultured under the following conditions: (1) standard culture conditions, (2) hypoxia (2% O_2_/5% CO_2_/balanced N_2_), (3) hypoxia with the p53 stimulator nutlin-3 (10 µM), and (4) hypoxia with the p53 inhibitor pifithrin-α (10 µM). After 48 hours of incubation, the cell lysates were collected and stored at −70°C for further processing.

### Transfection of Small Interfering RNAs (siRNAs) Against Microtubule-associated Protein Light Chain 3B (LC3B), Beclin-1, Damage-regulated Autophagy Modulator (DRAM), Bcl-2, and Bax into Cytotrophoblasts

Specific siRNAs for human LC3B (MAP LC3β, sc-43390), beclin-1 (sc-29797), DRAM (sc-96209), Bcl-2 (sc-61899), and Bax (sc-29212) as well as control siRNA (sc-44230 and sc-44231), were purchased from Santa Cruz Biotechnology (Santa Cruz, CA). Transfection of siRNA was performed using the Lipofectamine 2000 transfection reagent (Invitrogen) according to the manufacturers’ instructions. The transfection of siRNA and treatment with hypoxia were performed under serum deprivation conditions, as previously detailed [19]. The siRNA/transfection reagent mixture was overlaid on the cells and incubated at 37°C in a humidified CO_2_ incubator. After 6 hours, the overlaid siRNA/transfection reagent mixture was removed and replaced with serum-free cytotrophoblast culture medium and incubated in 2% O_2_/5% CO_2_/balanced N_2_ for 48 hours. After treatment, cell viability was estimated using the Trypan blue exclusion assay, and the cell lysates were collected and stored at −70°C for further processing. Control siRNAs, each containing a scrambled sequence that will not lead to the specific degradation of any known cellular mRNA, were used as negative controls.

**Figure 3:**
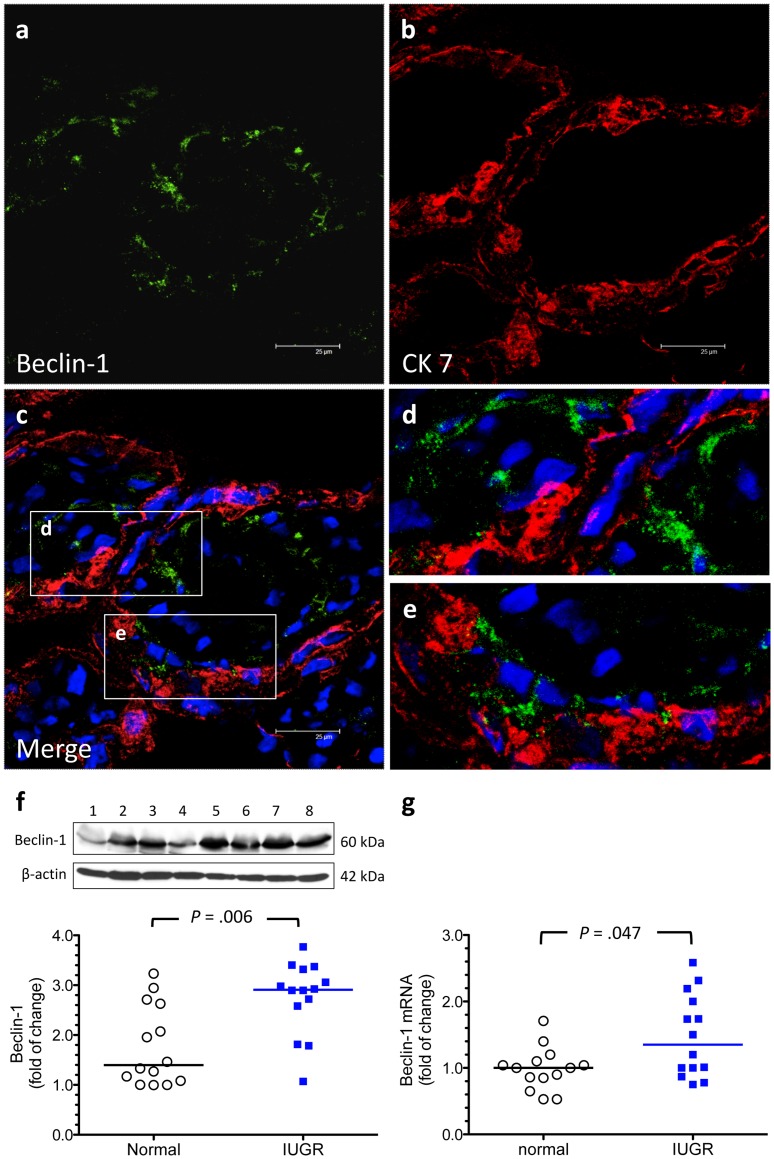
Increased beclin-1 levels in the placentas from pregnancies complicated by IUGR. (a–e) Formation of beclin-1-punctae was noted in the trophoblast layer and some stromal cells in the villous tissues from women with IUGR. Scale bar  = 25 µm. (f, g) There was a significant increase in the placental levels of beclin-1 protein and mRNA in women with pregnancies complicated by IUGR (n = 14) compared with those with normal pregnancies (n = 14). Lanes 1–4, normal placental samples; lanes 5–8, IUGR placental samples. Horizontal bars represent the median values. *P* values were based on the Mann-Whitney *U*-test.

### Immunofluorescence

Immunofluorescence was used to determine the localization of LC3B, beclin-1, DRAM, and cytokeratin 7 in the placenta of normal term and IUGR pregnancies, as previously described [20]. After blocking any non-specific binding, 5-µm cryosections were incubated with the following primary antibodies: rabbit anti-human LC3B (1∶50; catalogue no. #2775S, Cell Signaling, Danvers, MA), rabbit anti-human beclin-1 (1∶100; catalogue no. #3738S, Cell Signaling), rabbit anti-human DRAM (1∶100; catalogue no. ab68987, Abcam, Cambridge, UK) polyclonal antibodies, and a mouse anti-human cytokeratin 7 monoclonal antibody (clone OV-TL12/30, 1∶100; DakoCytomation, Glostrup, Denmark) at 4°C overnight. After washing, the sections were incubated with a cocktail of Alexa Fluor 488-conjugated goat anti-rabbit IgG and Alexa Fluor 594-conjugated goat anti-mouse IgG (1∶200; Molecular Probes; Life Technologies, Grand Island, NY) at room temperature for 1 hour, mounted with Vectashield-DAPI (Vector Laboratories, Burlingame, CA, USA), and observed on a Leica TCS-SP2 confocal microscope (Leica Microsystems, Manheim, Germany). The negative control condition used non-immune rabbit IgG or mouse isotypic IgG instead of the primary antibody.

**Figure 4:**
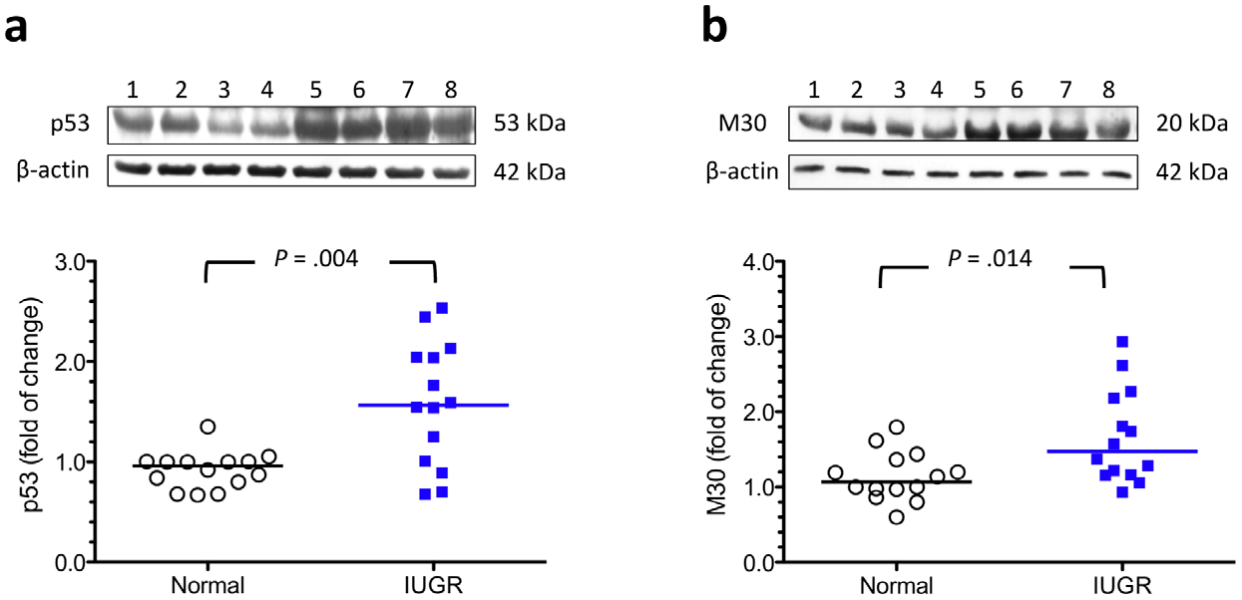
Increased p53 and M30 levels in the IUGR placentas. Increased levels of p53 (a) and M30 (b), a cytokeratin 18 neoepitope produced downstream by caspase proteolytic action, were noted in the placentas from IUGR (n = 14) compared with those from normal pregnancies (n = 14). Lanes 1–4, normal placental samples; lanes 5–8, IUGR placental samples. Horizontal bars represent the median values. *P* values were based on the Mann-Whitney *U*-test.

### Western Blotting

Western blotting was performed as previously detailed [21]. After individual experiment, Fifty to one hundred micrograms of cytosolic or nuclear proteins was separated by 12% or 16% SDS-PAGE, transferred to nitrocellulose membranes, and probed with primary antibodies against human LC3B (1∶500; Cell Signaling), beclin-1 (1∶500; Cell Signaling), DRAM (1∶400; Abcam), p53 (1∶200; catalogue no. sc-126, Santa Cruz), M30 (1∶100; catalogue no. 12140322001, Roche), Bcl-2 (1∶100; catalogue no. sc-492, Santa Cruz), and Bax (1∶100; catalogue no. #2772S, Cell Signaling) at 4°C overnight. The relative intensity of protein signals were normalized to the corresponding β-actin (clone AC-15, 1∶10000 dilution; Sigma) or histone H1 (clone FL-219, 1∶100 dilution; catalogue no. sc-10806, Santa Cruz) density and quantified by densitometric analysis using Image J software (National Institutes of Health, Bethesda, MD; http://rsb.info.nih.gov/ij/).

### Enzyme-Linked Immunosorbent Assay (ELISA)

ELISA for M30 was performed using a commercially available kit (M30 CytoDeath ELISA, Peviva AB, Bromma, Sweden) according to the manufacturers’ instruction. Cell lysate samples were diluted to fall within the linear portion of their respective standard curves, as determined in preliminary studies. All samples and standards were assayed in duplicate. The concentration of each sample was interpolated from the corresponding standard curve and was expressed as units per microgram of cellular protein. The detection limit for this immunoassay was 60 mU/mL, and the calculated inter- and intra-assay coefficients of variation were less than 10%.

### Real-time Quantitative PCR

Real-time quantitative PCR analysis was performed as previously described [21]. Assay-on-Demand TaqMan primers and probes for human LC3B (Hs00797944_s1), LC3C (Hs01374916_m1), beclin-1 (Hs00186838_m1), DRAM1 (Hs00218048_m1), p53 (Hs00153349_m1), and Bax (Hs00751844_s1) were obtained from Applied Biosystems; 18S ribosomal RNA (Hs99999901_s1) was used as an endogenous control. Thermal cycling was initiated with a 2-minute incubation at 50°C, followed by a first denaturation step of 10 minutes at 95°C, 40 cycles at 95°C for 15 seconds each, and 60°C for 1 minute. All samples were analyzed in the same run, and each sample was run in triplicate. Relative quantification of LC3B, LC3C, beclin-1, DRAM, and Bax mRNA to 18S ribosomal RNA was calculated by the comparative threshold cycle method, as previously described [21].

### Transmission Electron Microscopy

Autophagy of villous tissues were confirmed by transmission electron microscopy as previously described [21].

**Figure 5:**
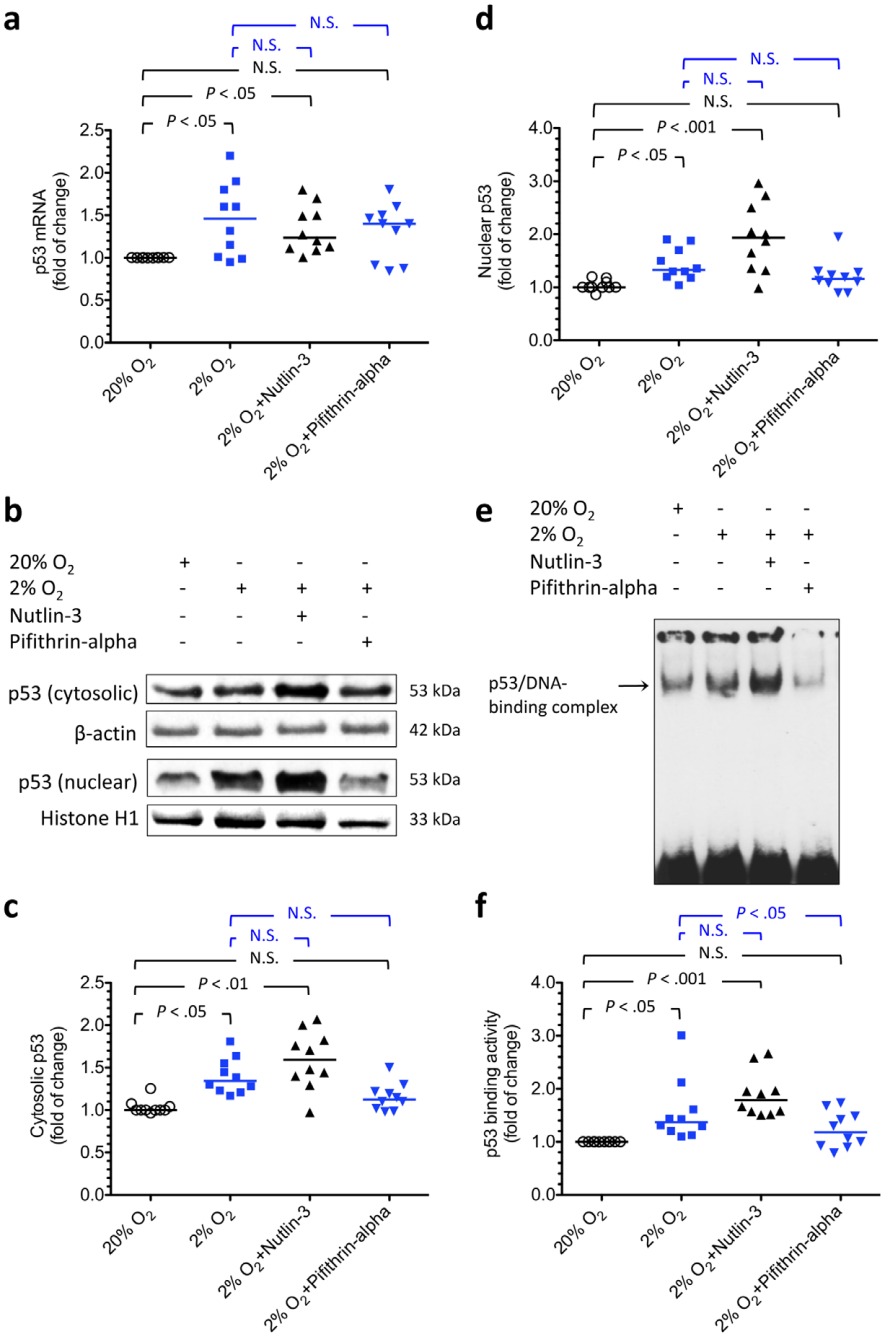
Effects of hypoxia, nutlin-3, and pifithrin-α on changes in p53 level or activity in cultured cytotrophoblasts. To verify the effects of hypoxia, nutlin-3, and pifithrin-α on changes in p53 level or activity, cytotrophoblasts were cultured under standard conditions, hypoxia (2% oxygen), or hypoxia with either nutlin-3 or pifithrin-α to regulate p53 activity. There was a significant increase in the expression of p53 mRNA (a) and protein (b–d) in cytotrophoblasts incubated under hypoxia with or without nutlin-3 compared with cells incubated under standard culture conditions. Hypoxia and nutlin-3 also led to an increase in p53 DNA-binding activity (e, f). In contrast, administering pifithrin-α under hypoxic conditions returned p53 mRNA and protein levels and p53 DNA-binding activity to those observed under standard conditions. (b) Representative immunoblots for cytosolic and nuclear p53 from cytotrophoblasts treated under different conditions are shown. β-actin and histone H1 were used to normalize for loading variability. (e) Representative gel shift analysis showing p53 DNA-binding activity under different experimental conditions. Horizontal bars represent the median values. *P* values were based on the Kruskal-Wallis test followed by Dunn’s multiple comparison test. A total of 10 individual experiments were performed.

### Electrophoretic Mobility Shift Assay (EMSA)

Nuclear extracts were prepared using a nuclear protein extraction reagent kit (Pierce Biotechnology, Rockford, IL). To evaluate p53 DNA-binding activity, EMSAs were performed using a commercial kit according to the manufacturer's protocol (p53 EMSA kit; Panomics, Inc., Fremont, CA). Briefly, a double-stranded biotin-labeled p53 oligonucleotide probe (5′- TACAGAACATGTCTAAGCATGCTGGGG -3′) was incubated with nuclear extracts in binding buffer and poly[d(I-C)] for 30 minutes on ice. Samples were separated by electrophoresis with 6% Tris-borate-EDTA (TBE) gels and transferred to nylon membranes. Oligonucleotides on the membranes were fixed for 3 minutes using a UV crosslinker. After incubating with blocking buffer at room temperature for 15 minutes, the membranes were reacted with streptavidin-conjugated horseradish peroxidase for another 15 minutes. After washing, the membranes were incubated with detection buffer at room temperature for 5 minutes, followed by incubation with chemiluminescent substrate solution for another 5 minutes. Shifted bands corresponding to the protein/DNA complexes were visualized relative to unbound double-stranded DNA after exposure to radiographic films. The specificity of the binding reaction was evaluated by adding a 50-fold excess of unlabeled probe to the protein/DNA reaction mixture, which competes with the labeled DNA probe for binding to the protein. For the supershift assay, a primary antibody against human p53 (1∶100; Santa Cruz) was incubated with the nuclear protein sample at 37°C for 1 hour before performing the binding reaction. Optical densities of the bands were quantified using Image J software.

**Figure 6:**
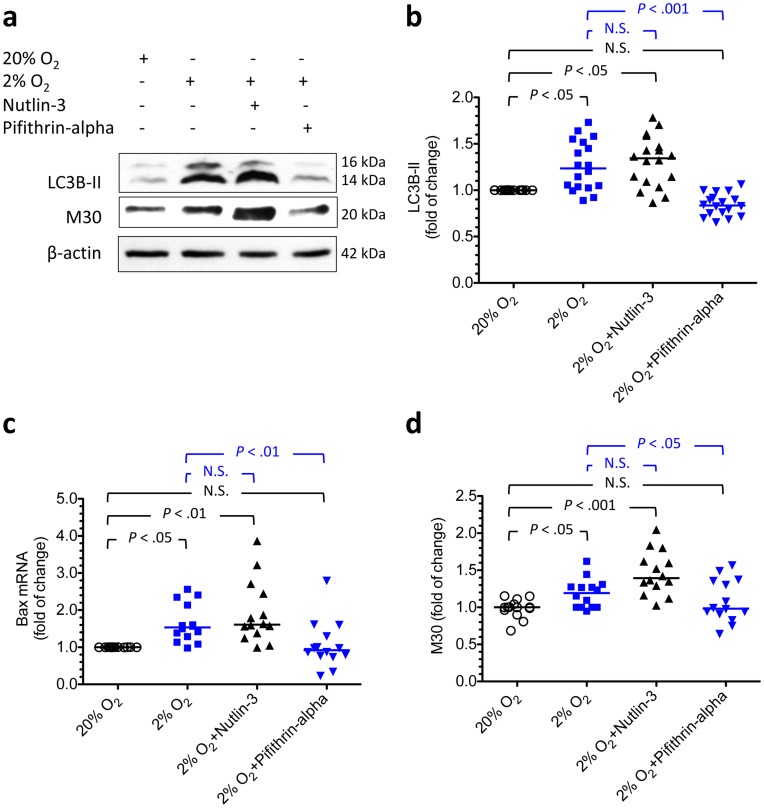
Effects of hypoxia, nutlin-3, and pifithrin-α on changes in LC3B-II and M30 levels and Bax mRNA in cultured cytotrophoblasts. To study the effect of hypoxia and the role of p53 in the regulation of autophagy and apoptosis, cytotrophoblasts were cultured under standard conditions, hypoxia (2% oxygen), or hypoxia with either nutlin-3 or pifithrin-α to regulate p53 activity. (a) Representative immunoblots for LC3B-II and M30 from cytotrophoblasts treated with different conditions are shown. β-actin was used to normalize for loading variability. (b) There was a significant increase in the levels of LC3B-II in cytotrophoblasts incubated under hypoxia with or without nutlin-3 compared with those incubated under standard culture conditions. These changes were associated with an increase in Bax mRNA (c) and M30 (d). In contrast, administration of pifithrin-α during hypoxia reduced LC3B-II, Bax mRNA, and M30 to levels similar to those observed under standard conditions. Horizontal bars represent the median values. *P* values were based on the Kruskal-Wallis test followed by Dunn’s multiple comparison test. At least 14 individual experiments were performed.

### Statistical Analyses

Data are presented as the means ± standard deviation or medians with the range when they were not normally distributed. Data were analyzed and plotted using Prism 5 for Mac OS X, version 5.0d (GraphPad Software, Inc., La Jolla, CA, USA). For comparisons among multiple groups, a one-way analysis of variance followed by Bonferroni’s test or Kruskal-Wallis test followed by Dunn’s multiple comparison test were used to determine significant differences. Differences between two groups were computed with the Mann-Whitney *U*-test. Statistical significance was accepted at *P*<0.05 for all comparisons.

**Figure 7:**
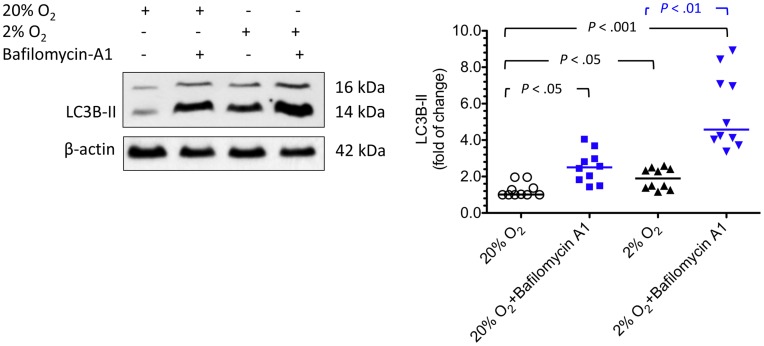
Bafilomycin A increased the level of LC3B-II in cytotrophoblasts under hypoxia. Cytotrophoblasts were incubated under standard or hypoxic (2% oxygen) conditions with or without bafilomycin A1, an inhibitor that inhibits degradation of autophagosome content. Treatment with bafilomycin A1 increased LC3B-II, indicating that these cells had a high basal rate of autophagy under hypoxia. Horizontal bars represent the median values. *P* values were based on the Kruskal-Wallis test followed by Dunn’s multiple comparison test. A total of 10 individual experiments were performed.

## Results

### Increased Autophagic Changes in the Placentas from Pregnancies Complicated by IUGR and PE+IUGR Compared with those From Normal Pregnancies

Microtubule-associated protein light chain 3 (LC3) is synthesized as proLC3, and autophagy-related protein 4 (Atg4) protease processes this precursor into LC3-I, with an exposed carboxy-terminal glycine. Upon induction of autophagy, the exposed glycine of LC3-I is conjugated to the highly lipophilic phosphatidylethanolamine (PE) moiety by Atg7 and Atg3 to generate LC3-II [22]. The PE group promotes integration of LC3-II into lipid membranes at the phagophore and autophagosomes. To date, LC3-II is the only well-characterized protein that is specifically localized to autophagic structures throughout the process from phagophore to lysosomal degradation [23].

The human LC3 family is composed of three isoforms, LC3A–C, with LC3B and LC3C transcription being noted in the human placenta [21]. Among these three isoforms, LC3B has the widest specificity, and only LC3B-II is correlated with increased levels of autophagic vesicles [24]. Therefore, LC3B-II is commonly used as a marker of autophagy [25].

Using an antibody that detects endogenous levels of total LC3B protein and has a stronger reactivity with the type II form of LC3B, immunofluorescence and Western blot techniques were performed to evaluate changes in autophagy between placentas from women with normal pregnancies and those with pregnancies complicated by IUGR, PE, or PE+IUGR. Compared with placentas from normal pregnancies, increased formation of LC3B-punctae in the trophoblasts and stromal cells and significantly higher levels of LC3B-II were noted in the placentas from pregnancies complicated by IUGR and PE+IUGR (Figure 1). There was, however, no difference in the levels of placental LC3B-II between women with normal pregnancies and those with pregnancies complicated by PE only.

**Figure 8:**
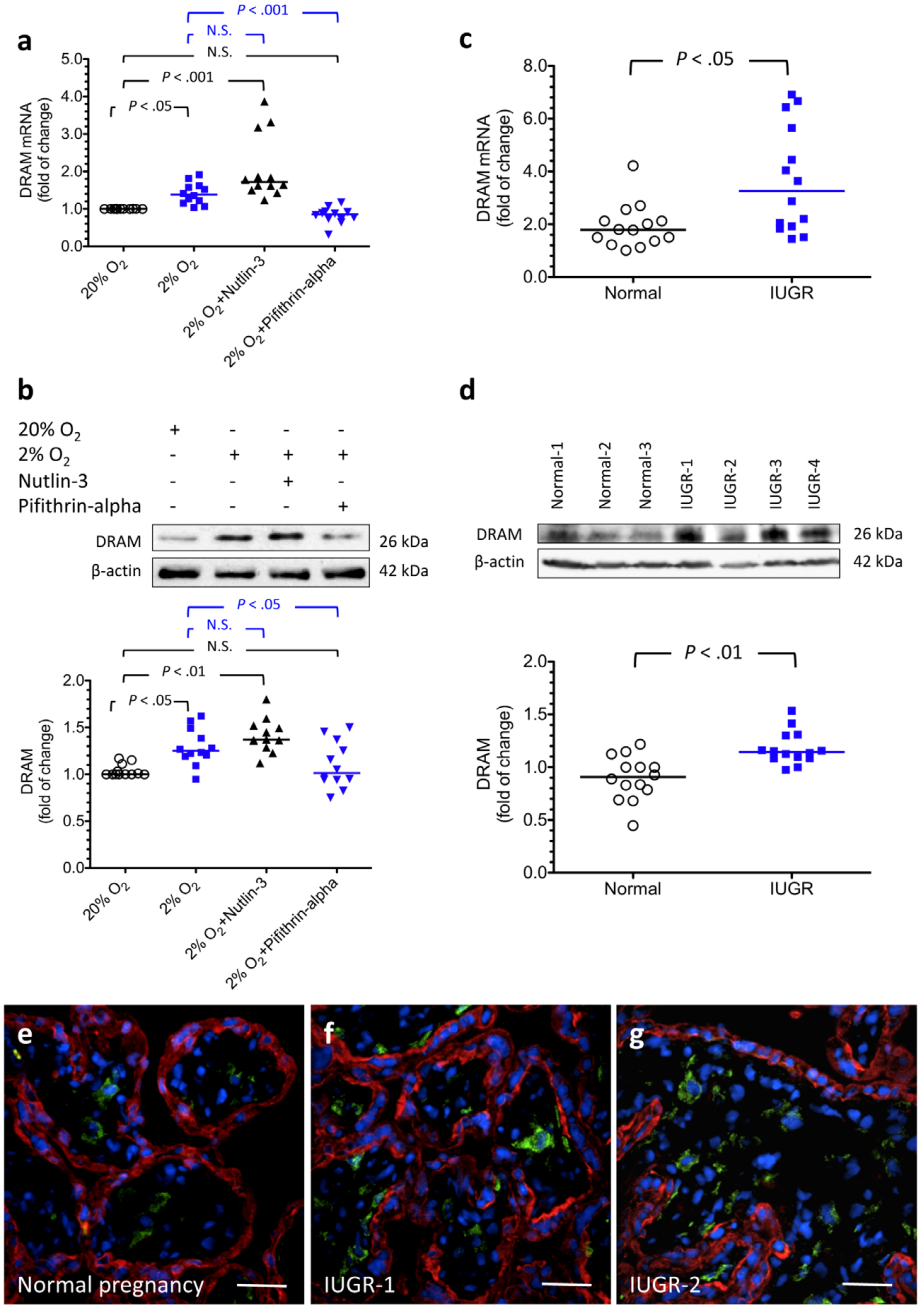
Increased DRAM mRNA expression and protein in trophoblasts under hypoxia and in IUGR placentas. (a, b) Hypoxia increased DRAM mRNA and protein levels in cytotrophoblasts. The levels of DRAM mRNA and protein increased further with administration of nutlin-3, while incubation with pifithrin-α reduced the changes. Horizontal bars represent the median values. *P* values were based on the Kruskal-Wallis test followed by Dunn’s multiple comparison test. A total of 12 individual experiments were performed. (c, d) In parallel, there were significantly higher levels of DRAM mRNA and protein in villous samples from women with IUGR (n = 14) compared with those from normal pregnant women (n = 14). Horizontal bars represent the median values. *P* values were based on the Mann-Whitney *U*-test. (e–g) Immunofluorescent labeling for DRAM (green) and cytokeratin 7 (red) in placental samples from a woman with a normal term pregnancy and two women with pregnancies complicated by IUGR. Note that DRAM was mainly localized at stromal cells and, to a lesser extent, the cytotrophoblasts. Compared with placentas of normal pregnancy, there were more DRAM immunofluoresence-positive cells in the villous tissues of IUGR. The sections were stained with DAPI (blue) to highlight all nuclei. Scale bar  = 50 µm.

Ultrastructural assessment using transmission electron microscopy revealed autophagic vacuoles in the trophoblast layer of placentas from pregnancies complicated by IUGR and PE+IUGR (Figure 2). These autophagic vacuoles contain intracytoplasmic organelles, such as mitochondria. Furthermore, placentas from pregnancies complicated by IUGR or PE+IUGR were more often associated with dysmorphism of the intracytoplasmic organelles in the trophoblast. These included loss of mitochondrial integrity and dilatation of cisternae of the rough endoplasmic reticulum.

**Figure 9:**
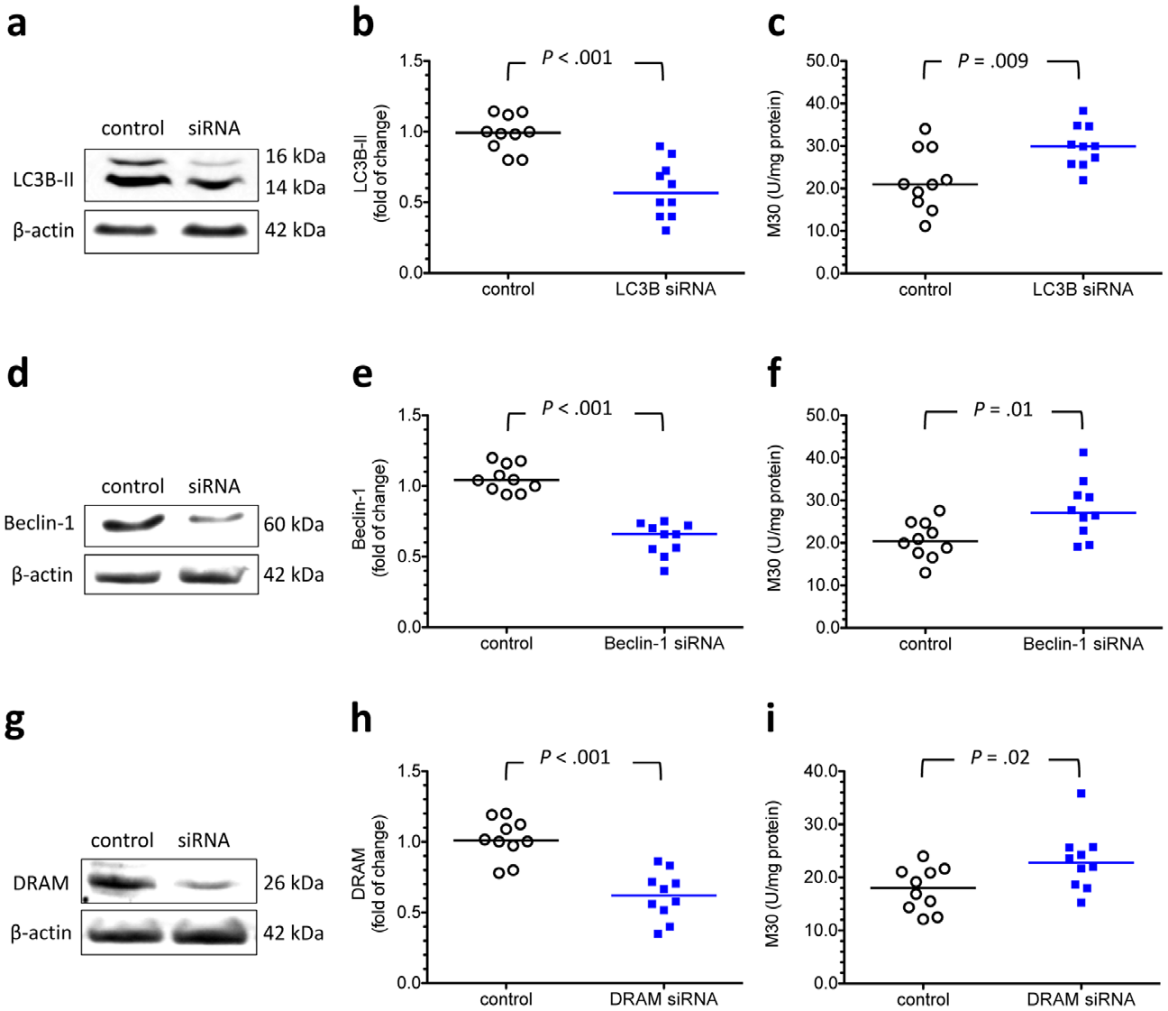
Effects of reducing the transcription of LC3B, beclin-1 and DRAM on the levels of M30 in cytotrophoblasts under hypoxia. To study the relationship between autophagy and apoptosis in cytotrophoblasts under hypoxia, the cells were transfected with LC3B, beclin-1 and DRAM-specific siRNA, and the levels of M30 were measured after 48 hours of incubation at 2% oxygen. (a, d, e) Representative immunoblots for LC3B-II, beclin-1, and DRAM from cytotrophoblasts transfected with or without specific siRNA are shown. β-actin was used to normalize for loading variability. (b, e, h) Compared with the control groups, cytotrophoblasts transfected with specific siRNAs had a 40–45% reduction in the levels of corresponding protein. (c, f, i) Compared with the control groups, cytotrophoblasts transfected with LC3B, beclin-1 or DRAM siRNA had higher levels of M30. Horizontal bars represent the median values. *P* values were based on the Mann-Whitney *U*-test. For each siRNA study, 10 individual experiments were performed.

Because there was no difference in the levels of placental LC3B-II between women with normal or PE pregnancies and because accumulating evidence suggests that PE and PE+IUGR may have different pathophysiologies [8], [26], we focused our subsequent investigations on the differences in autophagy between women with normal pregnancies and those with pregnancies complicated by IUGR only.

Beclin-1 is a part of an early complex that promotes synthesis and growth of pre-autophagosomal membranes [25]. Similarly, increased formation of beclin-1-punctae was noted in the trohoblast layer and some stromal cells in the villous tissues from women with IUGR. There was also a significant increase in the placental levels of beclin-1 mRNA and protein in IUGR pregnancies when compared with normal pregnancies (Figure 3). Although increased LC3B-II levels were noted in the IUGR placentas compared with normal placentas, there was no difference in the expression of LC3B and LC3C mRNA between these two groups (Figure 1).

**Figure 10:**
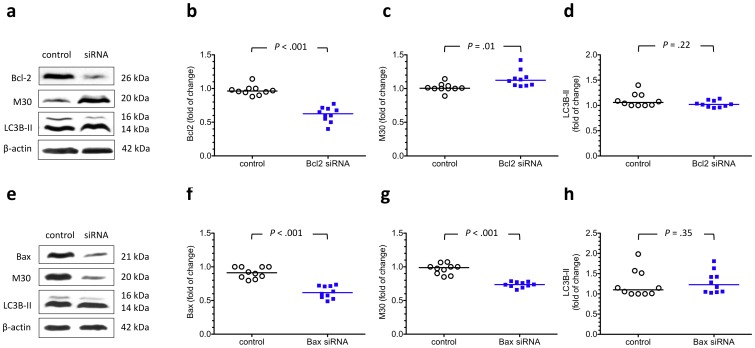
Effects of reducing the transcription of Bcl-2 and Bax on the levels of M30 and LC3B-II in cytotrophoblasts under hypoxia. To study the relationship between autophagy and apoptosis in cytotrophoblasts under hypoxia, the cells were transfected with Bcl-2- and Bax-specific siRNA, and the levels of M30 and LC3B-II were measured after 48 hours of incubation at 2% oxygen. (a) Representative immunoblots for Bcl-2, M30, and LC3B-II from cytotrophoblasts transfected with or without Bcl-2 siRNA are shown. Compared with the control groups, cytotrophoblasts transfected with Bcl-2 siRNA had a nearly 50% reduction in the levels of Bcl-2 protein (b) but a higher level of M30 (c). There was no difference in the levels of LC3B-II between the two groups (d). (e) Representative immunoblots for Bax, M30, and LC3B-II from cytotrophoblasts transfected with or without Bax siRNA are shown. Compared with the control groups, cytotrophoblasts transfected with Bax siRNA had significantly lower levels of Bax (f) and M30 (g) under hypoxia. There was no difference in the levels of LC3B-II between the two groups (h). β-actin was used to normalize for loading variability. Horizontal bars represent the median values. *P* values were based on the Mann-Whitney *U*-test. For each siRNA study, 10 individual experiments were performed.

### Increased p53 and M30 Levels in the IUGR Placentas Compared with those from Normal Pregnancies

As shown in Figure 4, there were significantly higher levels of p53 in the IUGR placentas compared with normal placentas. Similarly, increased apoptosis, as demonstrated by a significantly higher level of a cytokeratin 18 neoepitope (M30), which was produced downstream from caspase proteolytic action [27], was noted in IUGR placentas.

### Association between Changes in p53, Apoptosis and Autophagy in Cytotrophoblasts during Hypoxia

To study the role of p53 in the regulation of apoptosis and autophagy in trophoblasts, cytotrophoblasts were cultured under standard conditions (5% CO_2_ with balanced air), hypoxia (2% oxygen), or hypoxia with concomitant administration of nutlin-3 or pifithrin-α, and the levels of M30 and LC3B-II were compared. Nutlin-3 inhibits the interaction between Mdm2 and p53 and stabilizes the activity of p53, while pifithrin-α is a small molecule that inhibits the transcriptional activity of p53.

We first verified the effects of hypoxia, nutlin-3, and pifithrin-α on changes in p53 level or activity in cytotrophoblasts. As shown in Figure 5, there was a significant increase in the expression of p53 mRNA (Figure 5a) and protein (Figures 5b–5d) in cytotrophoblasts incubated under hypoxia with or without nutlin-3 compared with cells incubated under standard culture conditions. Hypoxia and nutlin-3 also led to an increase in p53 DNA-binding activity (Figures 5e and 5f). In contrast, administering pifithrin-α under hypoxic conditions returned p53 mRNA and protein levels and p53 DNA-binding activity to those observed under standard conditions.

After confirming the effects of hypoxia, nutlin-3, and pifithrin-α on p53 levels and activity, we next studied the impact of these experimental conditions on the autophagic and apoptotic changes in cytotrophoblasts. As shown in Figure 6, there was a significant increase in the levels of LC3B-II in cytotrophoblasts incubated under hypoxia with or without nutlin-3 compared with cells incubated under standard culture conditions. Hypoxia and nutlin-3 also caused an increase in the levels of Bax mRNA (a pro-apoptotic gene regulated by p53) and M30. In contrast, administrating pifithrin-α under hypoxic conditions returned LC3B-II, Bax mRNA, and M30 levels to those observed under standard conditions. These results imply that p53 may play a role in regulating the autophagy and apoptosis of cytotrophoblasts under hypoxic conditions.

### Bafilomycin A Increased the Level of LC3B-II in Cytotrophoblasts under Hypoxia

Increased LC3B-II levels can be associated with either enhanced autophagosome synthesis or reduced autophagosome turnover, probably due to delayed trafficking to the lysosomes, reduced fusion between compartments or impaired lysosomal proteolytic activity [25]. To better interpret changes in the levels of LC3B-II under hypoxia, cytotrophoblasts were incubated with 2% oxygen with or without bafilomycin A1, an inhibitor that inhibits autophagosome content degradation. As shown in Figure 7, treatment with bafilomycin A1 increases the amount of LC3B-II, indicating that these cells had a high basal rate of autophagy under hypoxia.

### Increased DRAM mRNA Expression and Protein in Trophoblasts under Hypoxia

Previous reports showed that p53 may induce and regulate autophagy by activating autophagy inducers, such as DRAM, which encodes a lysosomal protein [28]. We therefore investigated whether a similar mechanism exists in cytotrophoblasts treated with hypoxia. As shown in Figure 8, hypoxia increased the levels of DRAM mRNA and protein compared with standard culture conditions. DRAM mRNA and protein levels further increased in the presence of nutlin-3, while incubation with pifithrin-α reduced the levels. These results suggest that activation of DRAM is a possible mechanism underlying the effect of p53 on the regulation of autophagy in trophoblasts under hypoxia.

### Increased DRAM mRNA and Protein in IUGR Placentas Compared with those from Normal Pregnancies

To further verify the role of DRAM in the pathophysiology of IUGR, we studied the distribution of DRAM in the placentas and compared the placental levels of DRAM mRNA and protein between women with normal or IUGR pregnancies (Figure 8). DRAM was mainly localized in stromal cells and, to a lesser extent, the cytotrophoblasts. There were significantly higher levels of DRAM mRNA and protein in villous samples from women with IUGR compared with those from normal pregnant women.

### The Association between Autophagy and Apoptosis in Ctotrophoblasts Treated with Hypoxia

To investigate the relationship between autophagy and apoptosis, we knocked down the expression of LC3B, beclin-1 and DRAM mRNA using siRNAs and measured the M30 levels after 48 hours of incubation at 2% oxygen. Compared with the controls, cytotrophoblastics transfected with LC3B, beclin-1 or DRAM specific siRNAs had higher levels of M30 (Figure 9). However, there was no significant difference in the levels of LC3B-II between cytotrophoblasts transfected with specific siRNAs against apoptosis-related proteins, such as Bcl-2 and Bax, and the hypoxic controls (Figure 10). These findings indicate that autophagy may protect cytotrophoblasts from apoptosis induced by hypoxia.

## Discussion

Our study shows (1) increased autophagy in villous tissues from women with pregnancies complicated by IUGR or PE+IUGR but no difference in the placental levels of LC3B-II between women with normal pregnancies and those with PE only; (2) increased p53 and caspase-cleaved cytokeratin 18 (M30) in the IUGR placentas; (3) that cytotrophoblasts cultured under hypoxia (2% oxygen) in the presence or absence of nutlin-3 (a p53 activity stimulator) had higher levels of LC3B-II and M30 proteins and increased Bax mRNA expression compared with controls cultured under standard conditions, while administration of pifithrin-α (a p53 activity inhibitor) during hypoxia resulted in LC3B-II and M30 protein and Bax mRNA levels that were similar to those of the control groups; (4) an increase in LC3B-II levels when cytotrophoblasts were incubated with hypoxia and bafilomycin A1, which indicates that these cells had a high basal rate of autophagy under hypoxia; (5) increased levels of DRAM mRNA and protein in IUGR placentas; (6) increased expression of DRAM mRNA and protein in cytotrophoblasts when incubated with hypoxia in the presence or absence of nutlin-3 compared with the controls maintained under standard conditions, whereas administration of pifithrin-α during hypoxia reduced DRAM expression; (7) higher levels of apoptosis in cytotrophoblasts transfected with LC3B, beclin-1 or DRAM siRNA compared with untreated hypoxic cells; and (8) no significant difference in the levels of LC3B-II between cytotrophoblasts transfected with siRNAs against Bcl-2 and Bax, and the hypoxic controls. Together, these results suggest that autophagy and apoptosis play important roles in the pathophysiology of IUGR. Furthermore, the p53 pathway is involved in regulating autophagy and apoptosis in hypoxic trophoblasts.

The significance of autophagy in aging, infectious diseases, and neurodegenerative processes has become increasingly recognized; however, its role in the placental development and functions has not yet been elucidated. In association with apoptosis, autophagy has been found to be involved in the process of membrane rupture of human amnion in term gestation [29]. Furthermore, autophagy-related proteins, such as LC3 and beclin-1, have been demonstrated in the villous trophoblast through gestation, and increased levels of LC3-II were noted in placentas from pregnancies complicated by severe PE compared with those from normal pregnancies [30]. Recently, Signorelli and co-workers found that autophagy is more prominent in placentas obtained from cesarean sections than those from vaginal delivery, and there is an inverse relationship between the extent of autophagy and umbilical arterial glucose concentration [16]. Our recent work shows that there is a differential change in the autophagy of trophoblast between constant oxygen condition and hypoxia-reoxygenation [21]. Here, we extended these findings by demonstrating increased autophagy in IUGR placentas. These observations indicate that autophagy may contribute to placental development and pathological complications, such as PE and IUGR.

In this study, we found increased LC3B-II in the villous tissues of women with IUGR and PE+IUGR compared with women with normal pregnancies, while there was no difference in the levels of LC3B-II between normal pregnant women and women with PE only. This result is somewhat different though not necessarily inconsistent from a previous study [30], which is likely because we sub-classified our PE patients into those with maternal syndrome only and those with the more severe form having both maternal and fetal manifestations. Oh and colleagues included patients with IUGR as one of their criteria of severe PE, and the median birth weight in their severe PE patients was significantly lower than that of normal pregnant women (1671 g vs. 3265 g). As a result, the difference in the placental levels of LC3B-II between their control subjects and those with severe PE is likely caused by IUGR rather than PE. Accumulating evidence suggests that PE and PE+IUGR may have different etiologies and pathophysiologies [8], [26]. Therefore, we believe that separating these two groups is mandatory when studying the association between autophagy and the development of PE or IUGR.

Our study shows that women with IUGR had higher placental levels of LC3B-II compared with normal pregnant women, while similar levels of LC3B and LC3C mRNA were noted between these two groups of women. Explanations for this discrepancy are not clear; however, these results suggest that regulation of LC3B in IUGR placentas may occur at the posttranslational level. As mentioned previously, cytosolic LC3B-I is transformed to the PE-conjugated, membrane-bound form LC3B-II via a process catalyzed by autophagy-related proteins Atg7 and Atg3. It is possible that increased LC3B-II in IUGR placentas was caused by a change in these proteases with defective placentation. Indeed, upregulation of Atg7 and Atg3 has been noted during hypoxia or ischemia in other organ systems [31], [32].

Autophagy has been suggested to be a cell survival or cell death response and there is increasing evidence showing the existence of cross-talk between apoptosis and autophagy [2]. Consistent with previous reports [11], [33], we found that hypoxia caused additional apoptotic changes, including increased expression of Bax mRNA and caspase-cleaved cytokeratin 18 in trophoblasts. At the same time, these changes were associated with increased levels of autophagy-related proteins, such as LC3B-II, beclin-1 and DRAM. Trophoblast autophagy may suggest a causal relationship between autophagy and cell death. Alternatively, it may represent an adaptive reaction to support cell survival under hypoxic stress. In the present study, cytotrophoblasts transfected with LC3B, beclin-1 or DRAM siRNA had higher levels of M30 compared with the controls, indicating a protective role for autophagy against hypoxia-induced trophoblast apoptosis. Interestingly, knocking down the transcription of Bcl-2 and Bax did not cause any significant change in the level of LC3B-II in cytotrophoblasts under hypoxia. Further studies are needed to clarify the interaction between autophagy and apoptosis in the human placenta in response to different stress stimuli.

The significance of increased autophagy in IUGR placentas is unclear. Possible roles include maintenance of bioenergic homeostasis and clearance of damaged organelles. Compared with normal pregnancies, increased activation of mitochondrial apoptotic pathway [9], [11] and aggravation of endoplasmic reticulum stress [34] were noted in the villous trophoblasts from IUGR pregnancies. These changes may impair energy homeostasis and protein synthesis in the placenta and, thus, dysfunction of the placenta [8]. Autophagy may help the trophoblasts adapt to these disturbances to generate intracellular nutrients and energy and to remove damaged mitochondria and rough endoplasmic reticulum.

Broad and Keverne recently proposed a protective role of placental autophagy for fetal brain development during nutrient deprivation. They found that Peg3, a maternally imprinted gene, helps regulate synchronized expression of genes involved in the development of the placenta and the fetal hypothalamus [35]. A 24-hour food deprivation significantly decreased Peg3 gene expression in the placenta but increased expression in the hypothalamus. This biased change to gene dysregulation in the placenta is linked to autophagy and ribosomal turnover, which sustain nutrient supply for the developing hypothalamus. These results indicate that changes in gene expression brought about by food deprivation and suggest that the fetal genome is maintained throughout hypothalamic development at a cost to the placenta.

Similar to prior studies [11], [12], we found increased levels of p53 and apoptosis in IUGR placentas. We further extended these findings to show that p53 activation is involved in the regulation of autophagy in trophoblasts under hypoxia. One possible p53-mediated mechanism to regulate autophagy is through transcription of autophagy inducers, such as DRAM. Consistent with these findings, increased levels of DRAM mRNA and protein were noted in IUGR placentas. Moreover, DRAM-specific siRNA increased the vulnerability of cytotrophoblasts to hypoxic insult, suggesting that p53-induced autophagy is a negative regulator of p53-induced apoptosis. Together, these results indicate a pivotal and complex function of p53 in regulating trophoblast turnover in response to hypoxic stress. Notably, the role of p53 is further complicated by its cellular localization-dependent effect on the induction of autophagy [13], with cytosolic p53 inhibiting autophagy and nuclear p53 inducing and regulating autophagy. Further experiments are needed to study whether similar mechanisms exist in the human placenta.
